# Twenty-Four Hour Ambulatory Blood Pressure Monitoring in Offspring Conceived Through Assisted Reproductive Technologies

**DOI:** 10.3390/children12040507

**Published:** 2025-04-15

**Authors:** Felix Sebastian Oberhoffer, Pengzhu Li, Magdalena Langer, Theresa Vilsmaier, Marie Kramer, Franziska Sciuk, Brenda Kolbinger, André Jakob, Nina Rogenhofer, Robert Dalla-Pozza, Christian Thaler, Nikolaus Alexander Haas

**Affiliations:** 1 Division of Pediatric Cardiology and Intensive Care, University Hospital, LMU Munich, 81377 Munich, Germany; pengzhu.li.extern@med.uni-muenchen.de (P.L.); magdalena.langer@campus.lmu.de (M.L.); marie.kramer@campus.lmu.de (M.K.); franzisciuk@web.de (F.S.); brenda.kolbinger@t-online.de (B.K.); andre.jakob@med.uni-muenchen.de (A.J.); robert.dallapozza@med.uni-muenchen.de (R.D.-P.); nikolaus.haas@med.uni-muenchen.de (N.A.H.); 2 Division of Gynecological Endocrinology and Reproductive Medicine, Department of Gynecology and Obstetrics, University Hospital, LMU Munich, 81377 Munich, Germanynina.rogenhofer@med.uni-muenchen.de (N.R.); thaler@med.uni-muenchen.de (C.T.)

**Keywords:** blood pressure, assisted reproductive technologies, offspring

## Abstract

Background: One in fifty infants is conceived through assisted reproductive technologies (ART). To date, data on the cardiovascular morbidity of ART individuals is ambiguous. This study investigated the vascular function of young ART subjects using 24 hour ambulatory blood pressure monitoring (24 h ABPM). Methods: ART subjects and spontaneously conceived controls matched in age as well as sex were enrolled. A 24 h blood pressure profile including pulse wave analysis was executed in all study participants. Blood pressure readings were assessed every 15 min during daytime and every 30 min during nighttime. The 24 h systolic/diastolic blood pressure (SBP/DBP) as well as central blood pressure, nocturnal blood pressure decrease, and 24 h pulse wave velocity (PWV) were analyzed. Results: A total of 41 ART individuals and 46 spontaneously conceived peers were included in the final analysis (mean age: 15.37 ± 5.46 years vs. 16.48 ± 5.23 years, *p* = 0.338). The 24 h SBP (112.74 ± 9.24 mmHg vs. 112.73 ± 6.70 mmHg, *p* = 0.997), 24 h DBP (65.61 ± 7.98 mmHg vs. 66.57 ± 7.03 mmHg, *p* = 0.550), 24 h central blood pressure, nocturnal blood pressure decrease, and 24 h PWV did not demonstrate significant differences between the ART and control group. Conclusions: In contrast to previous studies, no significant differences in 24 h blood pressure were demonstrated between ART subjects and spontaneously conceived peers. Hence, the results of this study do not indicate an unfavorable blood pressure profile in ART offspring. Larger multi-centric studies are needed to validate these results in the future.

## 1. Introduction

According to the World Health Organization (WHO), one in six individuals has experienced infertility at some stage in their lives [1]. Assisted reproductive technologies (ART), such as in vitro fertilization (IVF) or intracytoplasmic sperm injection (ICSI), have now been used for many decades to treat infertility [2]. Today, more than 8 million human beings worldwide have been conceived with the help of ART [3]. In Europe, approximately one in fifty infants is conceived through ART [2].

The cardiovascular morbidity of ART offspring has become of special interest in the past: multiple studies indicated distinct vascular alterations in ART minors including elevated blood pressure, pulse wave velocity (PWV), and carotid intima-media thickness, as well as decreased endothelial function [4,5,6]. In contrast, recent studies by Halliday et al. and our working group could not confirm the above-mentioned findings [7,8,9]. Currently, data on the cardiovascular function of ART offspring are ambiguous.

Twenty-four hour ambulatory blood pressure monitoring (24 h ABPM) is considered the gold standard for the detection of arterial hypertension, a major predictor for future cardiovascular events [10]. However, limited data on 24 h ABPM is available for the ART offspring cohort.

Hence, this study aimed to evaluate whether young ART individuals display significant differences in 24 h ABPM compared to spontaneously conceived peers.

## 2. Materials and Methods

### 2.1. Ethical Statement

This study was approved on the 27 December 2020 by the Ethics Committee of the Medical Faculty of LMU Munich (Ethikkommission der Medizinischen Fakultät der Ludwig-Maximilians-Universität München, Pettenkoferstraße 8a, 80336 Munich, Germany; approval number 20-0844) and was performed in accordance with the ethical standards of the Declaration of Helsinki. All participants gave their written informed consent to participate in the present study. For underage study participants, prior written informed consent was additionally obtained from parents or legal guardians.

### 2.2. Study Design and Study Population

The Munich heARTerY-study (Assisted Reproductive Technologies and their effect on heart and arterial function in Youth), a single-center observational cohort study, was conducted between May 2021 and March 2022. ART subjects were recruited in cooperation with the Division of Gynecological Endocrinology and Reproductive Medicine, Department of Obstetrics and Gynecology, University Hospital, LMU Munich (Munich, Germany). Age- and sex-matched spontaneously conceived peers were recruited via public calls within the greater Munich area.

### 2.3. Assessment of Anthropometric Variables

Bodyweight (kg), body height (cm), and body mass index (BMI, kg/m^2^) were recorded. The weight classification in underage participants was defined as follows: underweight if BMI < 10 percentile (P.), normal weight if BMI ≥ 10 percentile (P.) but < 90 percentile (P.), overweight if BMI ≥ 90 percentile (P.), obese if BMI ≥ 97 percentile (P.) [11]. For adult participants, the following weight classification was applied: underweight if BMI < 18.5 kg/m^2^, normal weight if BMI ≥ 18.5 kg/m^2^ but <25 kg/m^2^, overweight if BMI ≥ 25 kg/m^2^ but <30 kg/m^2^, obese if BMI ≥ 30 kg/m^2^.

### 2.4. Medical History, Course of Pregnancy and Birth, Maternal Educational Level, Clinical Examination

Study participants were questioned regarding their medical history, their smoking status, and their regular use of medications. Data on the course of pregnancy and birth were retrospectively gathered through clinical records and parental interviews, including birth weight (g), birth height (cm), gestation age (weeks), cases of multiple pregnancy, maternal age at birth (years), maternal BMI at conception (kg/m^2^), presence of gestational diabetes, and maternal blood pressure during pregnancy ≥ 140/90 mmHg. Additionally, maternal educational level was recorded according to the German educational system: no school leaving qualification (0), lower secondary school leaving certificate (1), intermediate secondary school leaving certificate (2), general qualification for university entrance (3), completed apprenticeship (4), and completed university degree (5).

### 2.5. Adherence to the Mediterranean Diet, Physical Activity, and Sedentary Behavior

The validated 14-item Mediterranean diet assessment tool by Martínez-González et al. was translated into German and applied for adult study participants to evaluate their adherence to Mediterranean diet [12]. For participants < 18 years, the KIDMED index by Serra-Majem et al. was translated into German and used [13]. A score ≥ 8 was considered a high adherence to the Mediterranean diet [12,13]. The German version of the Global Physical Activity Questionnaire developed by the WHO was selected to assess the level of physical activity in adult study participants [14]. Picture cards were presented for each activity domain [14]. Total and recreational metabolic-equivalent-(MET) min per week were calculated [14]. For adult participants, WHO physical activity recommendations were met if they achieved ≥ 600 total MET-min per week [14]. Moreover, the number of muscle-strengthening activities per week as well as the time spent with sedentary activities per day (min) were evaluated [14]. Study participants < 18 years were asked how much time they spent per day on moderate and/or vigorous physical activities. In addition, underage subjects were asked how many times vigorous, muscle-strengthening and/or bone-strengthening activities were executed per week. Picture cards were presented for each activity domain [15]. Minor subjects met the WHO recommendations if an average of ≥60 min per day was accomplished for moderate and/or vigorous activities, and if vigorous, muscle-strengthening, and/or bone-strengthening activities were performed ≥ 3 times per week [15]. Daily sedentary behavior (min) was additionally recorded [15].

### 2.6. Twenty-Four Hour Ambulatory Blood Pressure Monitoring

An automated oscillometric blood pressure device (Mobil-O-Graph^®^, IEM, Aachen, Germany) was used for 24 h ABPM. Cuff sizes were picked according to the participant’s right upper arm circumference. During daytime, the device performed measurements automatically every 15 min and during nighttime, every 30 min. Subjects were asked to record sleep and wake times on a separate protocol. The 24 h ABPM data were cleaned (e.g., measurement errors) and analyzed by a masked researcher. The study participants were included in the final analysis if ≥40 blood pressure measurements were successfully performed within 24 h and if ≥10 blood pressure measurements were successfully recorded during nighttime. The average for the following values was evaluated for daytime, nighttime, and the total 24 h period: systolic blood pressure (SBP, mmHg), diastolic blood pressure (DBP, mmHg), mean arterial pressure (MAP, mmHg), pulse pressure (PP, mmHg), central systolic blood pressure (cSBP, mmHg), central diastolic blood pressure (cDBP, mmHg), central pulse pressure (cPP, mmHg), PWV (m/s), and heart rate (HR, bpm). Dipping (%) was calculated for SBP and DBP as follows:

$$Dipping\left(\%\right)=\frac{\left(Mean\;Blood\;Pressure\;Daytime-Mean\;Blood\;Pressure\;Nightime\right)}{Mean\;Blood\;Pressure\;Daytime}\times 100$$

### 2.7. Statistical Analysis

Statistical analysis was performed utilizing SPSS 29 (Release Date 2023, IBM SPSS Statistics for Windows, version 29.0, IBM Corp., Armonk, NY, USA). The Shapiro–Wilk test, histograms, and QQ-plots were used to test the normality of the continuous parameters. Nominal data were compared using Pearson’s Chi-squared test and Fisher’s exact test. For continuous and normally distributed variables, the unpaired *t* test was utilized. If continuous data were non-normally distributed the Mann–Whitney *U* test was applied. Data were given as mean ± standard deviation (SD) for normally distributed continuous parameters and as the median (interquartile range, (IQR)) if non-normally distributed. A *p*-value < 0.05 was considered statistically significant.

## 3. Results

### 3.1. Patients’ Characteristics

In total, 70 ART subjects and 86 control subjects were enrolled in this study. Within the ART cohort, one patient was excluded from further analysis due to a history of T-cell lymphoma, one due to a history of heart surgery, and twenty-seven ART subjects were excluded due to insufficient 24 h ABPM data quality. Within the control group, 40 subjects were excluded due to insufficient 24 h ABPM data quality.

In total, 41 ART subjects (IVF, *n* = 11; ICSI, *n* = 29; gamete intrafallopian transfer, *n* = 1) and 46 control subjects were included in the analysis. Within the ART group, one subject had long QT syndrome, one had a bicuspid aortic valve, one had a questionable history of myocarditis, one had hypothyroidism, and one had a history of hypercholesterolemia. Four ART subjects were using oral contraceptives, one was using L-Thyroxine, and one was using methylphenidate. Within the control group, six subjects were taking oral contraceptives, and one subject was taking bisoprolol due to a chronic migraine.

ART subjects and controls did not differ significantly in age (15.37 ± 5.46 years vs. 16.48 ± 5.23 years, *p* = 0.338) and gender. Height was significantly lower in the ART individuals (156.45 ± 18.93 cm vs. 165.95 ± 15.73 cm, *p* = 0.012). Other anthropometric variables, including bodyweight, BMI, or weight classification, did not show significant differences between both groups. In addition, smoking status did not display significant differences between the ART and the control group.

ART subjects demonstrated a significantly lower birth weight and birth height compared to controls. Moreover, the prevalence of multiple pregnancy was significantly higher in the ART cohort. No significant differences were seen in gestational age, maternal age at birth, maternal BMI at conception, prevalence of gestational diabetes, prevalence of maternal blood pressure during pregnancy ≥ 140/90 mmHg, as well as maternal educational level. Table 1 summarizes the patients’ characteristics.

### 3.2. Adherence to the Mediterranean Diet, Level of Physical Activity, and Sedentary Behavior

No significant differences were found in terms of adherence to the Mediterranean diet and level of physical activity between both groups (Table 2). Minor ART participants engaged significantly less in sedentary behavior compared to their spontaneously conceived peers (Table 2).

### 3.3. Twenty-Four Hour Ambulatory Blood Pressure Monitoring

The blood pressure profile, including dipping, central blood pressure, and PWV, did not show significant differences between ART subjects and spontaneously conceived controls (Table 3). ART individuals demonstrated a significantly higher HR during daytime and for the total 24 h period (Table 3).

## 4. Discussion

In this study, 41 ART subjects and 46 controls were included. Both groups were matched by age and sex. Despite the height being significantly lower in ART subjects, both groups did not show significant differences in anthropometric variables. While the adherence to the Mediterranean diet and the physical activity level did not alter significantly between both groups, the underage ART group engaged significantly less in sedentary behavior. In contrast to previous reports, the 24 h blood pressure profile did not differ significantly between ART subjects and their spontaneously conceived peers. Further, HR was significantly higher in ART individuals.

### 4.1. Comparison to Previous Findings

The gold standard for identifying arterial hypertension, a major predictor of future cardiovascular events, is 24 h ABPM [10]. However, data on the 24 h blood pressure profile in ART subjects is sparse. To the best of our knowledge, this is the second study investigating the 24 h blood pressure profile in an ART cohort. A well-known study by Meister et al. assessed the 24 h blood pressure profile in 54 ART subjects and 43 controls (mean age: 16.5 ± 2.3 years vs. 17.4 ± 2.7 years) [4]. The authors were able to show a significantly higher 24 h SBP (119.8 ± 9.1 mmHg vs. 115.7 ± 7.0 mmHg, *p* = 0.02) as well as 24 h DBP (71.4 ± 6.1 mmHg vs. 69.1 ± 4.2 mmHg, *p* = 0.03) in the examined ART cohort [4]. In addition, more than 15% of ART subjects, but only 2.5% of controls, fulfilled the criteria for the diagnosis of arterial hypertension (*p* = 0.041) [4]. Moreover, ART subjects displayed a significantly lower endothelial function and a significantly increased carotid intima-media thickness [4]. In contrast to our study cohort, the subjects examined by Meister et al. were older, singletons, born at term, displayed normal birth weight, and did not differ in height [4]. Lifestyle factors (e.g., level of physical activity) were not addressed by the authors [4]. Therefore, the results of both studies are not fully comparable. The vascular function of our ART cohort was potentially positively influenced by a significantly lower height and sedentary behavior. Since their introduction in 1978, ART have made significant advancements in treatment protocols, culture conditions, quality control, as well as fertilization and pregnancy success rates [16,17,18]. Moreover, a significant decline in adverse perinatal outcomes has been noticed over the past few decades [19]. Therefore, the advancement of ART procedures over time could potentially explain the unaltered cardiovascular function displayed in more recent ART studies. In our study, HR was significantly higher in ART individuals, possibly due to a significantly lower height and a higher rate of adverse perinatal risk factors (e.g., prematurity, low birth weight) [20,21]. The significantly higher HR in ART individuals could also be a sign of sympathetic predominance, as shown by Mizrak et al. [22]. The authors investigated the cardiovascular autonomic nervous function in children conceived by ART and found a significantly lower heart rate variability and vagal modulation compared to controls [22].

As an increased HR is linked with a higher cardiovascular morbidity within the general population, longitudinal multi-center studies with a larger sample size are needed to validate the current results in ART offspring [23]. In accordance with Meister et al., a Chinese meta-analysis, including 2112 subjects conceived via IVF or ICSI as well as 4096 naturally conceived peers, displayed a significantly higher SBP and DBP in ART offspring [16].

In contrast, a study led by Halliday et al. did not find significant differences in vascular function (e.g., blood pressure, PWV, carotid intima-media thickness) between 193 adult ART subjects and 86 spontaneously conceived individuals [7]. Prior publications of our working group displayed an unaltered vascular (e.g., endothelial function, arterial stiffness, carotid intima-media thickness) as well as metabolic function (e.g., lipid profile, HbA1c) in the same ART cohort [8,9].

Similarly, large ART cohort studies did not observe an increased risk for the development of cardiovascular diseases or hospitalization due to cardiovascular diseases [24,25]. In addition, a recent meta-analysis by Yeung et al., involving 5986 individuals conceived via ART and 41,496 subjects conceived spontaneously found no association between ART and offspring blood pressure [26].

To examine whether the vascular system of ART individuals ages more profoundly than that of spontaneously conceived controls, studies with a longitudinal study design are required. In addition, multi-center studies with a larger sample size are required for a more precise cardiovascular risk stratification of the ART cohort.

### 4.2. Pathophysiological Considerations

ART is linked with pregnancy complications and adverse perinatal outcomes (e.g., maternal hypertension, gestational diabetes, prematurity, and small for gestational age) that are thought to negatively influence the offspring’s vascular function [27]. Further, as male and female infertility is associated with an elevated cardiovascular morbidity, individuals conceived via ART may inherit certain cardiovascular risk factors [28,29]. Interestingly, recent publications of our working group could not demonstrate an elevated cardiovascular morbidity (e.g., blood pressure, PWV, carotid intima-media thickness, lipid profile) in parents with ART history [30,31]. The literature suggests that the ART procedure itself, female infertility, advanced maternal age, pregnancy complications, and perinatal risk factors might elevate oxidative stress levels [32,33]. Increased oxidative stress levels during the ART procedure could result from various factors, including cryopreservation, fluctuations in pH and temperature, culture media composition, and the absence of natural antioxidant mechanisms [34]. Since the cardiovascular system is one of the first to form during embryonic development, it may be particularly vulnerable to negative environmental influences [32]. Oxidative stress can result in epigenetic changes and thus alterations of cardiovascular development [32]. In recent years, revised ART protocols (e.g., improved oocyte handling, reduced exposure to atmospheric oxygen, modified culture media) have contributed to the better control of oxidative stress levels, which could explain some inconsistencies found in the literature regarding the cardiovascular morbidity of ART offspring [33]. For the precise assessment of the pathophysiological pathways involved in the potentially increased cardiovascular morbidity of ART subjects, further experimental studies are required.

### 4.3. Strengths and Limitations

#### 4.3.1. Study Design

This was a single-center study including 41 ART study participants and 46 spontaneously conceived controls. Special emphasis was put on age- and gender-matching. Moreover, lifestyle factors were addressed. ART subjects with adverse perinatal conditions were intentionally enrolled to portray the true cardiovascular risk profile of this cohort. Excluding these individuals would have significantly lowered the sample size. ART subjects had significantly lower birth weight and height, as well as shorter overall height, and engaged significantly less in sedentary behavior. Moreover, the prevalence of multiple pregnancy was significantly higher in the ART group. Hence, the differences in patients’ characteristics and sedentary behavior between both groups may have altered the demonstrated vascular results. Further, this study did not account for potential differences between ART procedures (e.g., ICSI, IVF), which could have varying impacts on cardiovascular health. The sample size of the current study can be considered as relatively small, which may have limited the statistical power to detect subtle cardiovascular differences between both groups. Moreover, the study’s cross-sectional design limited the ability to capture long-term cardiovascular trends in ART individuals. Hence, multi-centric studies with a larger sample size and a longitudinal study design are required in the future to adjust for the above-mentioned confounders and to confirm the demonstrated results. In the meantime, ART individuals, particularly those with increased perinatal morbidity, could benefit from regular cardiovascular screening.

#### 4.3.2. Methods

Data regarding pregnancy and birth histories were retrospectively obtained from medical records and parent interviews. Missing and/or incomplete medical records for some participants resulted in a loss of information. In this study, 24 h ABPM data were recorded automatically by the blood pressure device and analysis was conducted by a masked researcher. To ensure high 24 h ABPM data quality, only study participants with ≥40 blood pressure measurements within 24 h and ≥10 blood pressure measurements during nighttime were included. This led to an exclusion of 27 ART subjects as well as 40 spontaneously conceived peers, and thus to a distinctly lower sample size. The Mobil-O-Graph^®^ (IEM, Rheinland, Germany) is recommended as a blood pressure device for clinical practice, as it complies with the European Society of Hypertension standards [34]. However, compared to other blood pressure devices, the Mobil-O-Graph^®^ is thought to underestimate markers of pulse wave analysis [35].

## 5. Conclusions

In this study, no significant differences in the 24 h blood pressure profile were observed between ART individuals and spontaneously conceived peers. Hence, unlike previous publications, the results of this study do not indicate an unfavorable blood pressure profile in ART offspring. In the future, multi-center studies with a longitudinal study design are needed for a more precise cardiovascular risk stratification of the ART cohort to confirm the demonstrated results.

## Figures and Tables

**Table 1:** Patients’ characteristics.

Variable	ART (*n* = 41)	Control (*n* = 46)	*p*-Value
Age (Years)	15.37 ± 5.46	16.48 ± 5.23	0.338
Female (*n* (%))	24 (58.5)	23 (50.0)	0.457
Bodyweight (kg)	50.01 ± 18.53	54.16 ± 15.12	0.254
Height (cm)	156.45 ± 18.93	165.95 ± 15.73	0.012 *
BMI (kg/m^2^)	19.53 ± 3.58	19.21 ± 2.85	0.644
Weight classification			
Underweight (*n* (%))	3 (7.32)	3 (6.52)	0.351
Normal weight (*n* (%))	34 (82.93)	42 (91.31)	
Overweight (*n* (%))	4 (9.75)	1 (2.17)	
Smoking (*n* (%))	3 (7.32)	1 (2.17)	0.339
Course of pregnancy and birth, maternal educational level			
Birth weight (g) ^1^	3000 (2310–3210)	3440 (3170–3716)	<0.001 ***
Birth height (cm) ^2^	50.00 (48.00–52.00)	52.00 (50.00–54.00)	0.005 **
Gestational age (weeks) ^3^	38.00 (36.00–39.00)	39.00 (38.00–40.00)	0.062
Multiple pregnancy (*n* (%))	14 (34.15)	1 (2.17)	<0.001 ***
Maternal age at birth (years) ^4^	34.41 ± 3.53	33.30 ± 3.98	0.179
Maternal BMI at conception (kg/m^2^) ^5^	22.49 (20.57–26.11)	21.22 (20.24–23.09)	0.153
Gestational diabetes (*n* (%)) ^6^	1 (3.13)	1 (2.70)	1.000
Maternal blood pressure during pregnancy ≥ 140/90 mmHg (*n* (%)) ^7^	0 (0)	0 (0)	-
Maternal educational level ^8^	4.00 (2.00–5.00)	5.00 (4.00–5.00)	0.918

ART, assisted reproductive technologies; BMI, body mass index. ^1^ A total of 39 ART subjects and 40 control subjects were included; ^2^ a total of 37 ART subjects and 40 control subjects were included; ^3^ a total of 36 ART subjects and 39 control subjects were included; ^4^ a total of 40 ART subjects were included; ^5^ a total of 25 ART subjects and 27 control subjects were included; ^6^ a total of 32 ART subjects and 37 control subjects were included; ^7^ a total of 14 ART subjects and 20 control subjects were included; ^8^ a total of 27 ART subjects and 31 control subjects were included. Data are presented as mean ± SD if normally distributed and as median (IQR) if non-normally distributed. * *p*-value < 0.05. ** *p*-value < 0.01. *** *p*-value < 0.001.

**Table 2:** Adherence to the Mediterranean diet, level of physical activity, and sedentary behavior.

Variable	ART (*n* = 41)	Control (*n* = 46)	*p*-Value
Adult study participants	*n* = 16	*n* = 20	
MEDAS	6.19 ± 2.37	7.30 ± 1.63	0.105
Total MET (min/week) ^1^	5303 ± 4377	4626 ± 2442	0.595
Recreational MET (min/week) ^2^	2220 (675–5160)	1800 (960–2850)	0.726
Muscle-strengthening activities (times/week) ^1^	1.00 (0.00–2.00)	2.00 (0.00–3.00)	0.548
Sedentary behavior (min/day)	440.63 ± 159.02	390.00 ± 131.31	0.303
Minor study participants	*n* = 25	*n* = 26	
KIDMED	5.92 ± 2.34	6.19 ± 2.14	0.666
Moderate and/or vigorous physical activities (min/day)	60.00 (60.00–105.00)	90.00 (60.00–135.00)	0.555
Vigorous, muscle-strengthening and/or bone-strengthening activities (times/week)	3.08 ± 1.63	3.58 ± 1.65	0.284
Sedentary behavior (min/day) ^3^	360.00 (360.00–420.00)	420.00 (420.00–480.00)	0.025 *

ART, assisted reproductive technologies; MEDAS, Mediterranean diet adherence score; MET, metabolic-equivalent; KIDMED, Mediterranean diet quality index for children and adolescents. ^1^ A total of 15 ART subjects were included; ^2^ a total of 14 ART subjects were included; ^3^ a total of 24 ART subjects were included. Data are presented as mean ± SD if normally distributed and as median (IQR) if non-normally distributed. * *p*-value <0.05.

**Table 3:** Twenty-Four Hour Ambulatory Blood Pressure Monitoring.

	Daytime			Nighttime			24 h Period		
	ART (*n* = 41)	Control (*n* = 46)	*p*-Value	ART (*n* = 41)	Control (*n* = 46)	*p*-Value	ART (*n* = 41)	Control (*n* = 46)	*p*-Value
SBP (mmHg)	117.12 ± 9.19	116.18 ± 7.06	0.593	102.96 ± 8.05	103.67 ± 6.13	0.641	112.74 ± 9.24	112.73 ± 6.70	0.997
SBP Dipping (%)	-			13.89 ± 5.69	12.22 ± 5.99	0.186	-		
DBP (mmHg)	69.60 ± 8.10	69.76 ± 7.27	0.922	56.97 ± 6.39	58.13 ± 6.31	0.397	65.61 ± 7.98	66.57 ± 7.03	0.550
DBP Dipping (%)	-			22.34 ± 8.55	20.46 ± 9.46	0.334	-		
MAP (mmHg)	91.37 ± 8.21	91.02 ± 6.86	0.830	78.05 ± 6.72	78.99 ± 5.80	0.486	87.20 ± 8.21	87.71 ± 6.59	0.751
PP (mmHg)	47.52 ± 5.23	46.42 ± 4.16	0.279	45.99 ± 5.12	45.54 ± 4.58	0.669	47.13 ± 4.93	46.16 ± 3.92	0.309
cSBP (mmHg) ^1^	102.31 ± 9.02	101.36 ± 6.98	0.586	93.85 ± 7.90	94.73 ± 7.09	0.590	99.44 ± 8.74	99.31 ± 6.67	0.942
cDBP (mmHg) ^1^	71.90 ± 8.11	72.10 ± 7.48	0.905	58.25 ± 6.71	59.04 ± 6.60	0.585	67.22 ± 8.33	68.12 ± 7.28	0.595
cPP (mmHg) ^1^	30.39 ± 4.16	29.27 ± 3.07	0.155	35.59 ± 4.68	35.69 ± 3.66	0.919	32.20 ± 4.07	31.19 ± 2.70	0.189
PWV (m/s) ^1^	4.74 ± 0.30	4.72 ± 0.27	0.762	4.40 ± 0.31	4.46 ± 0.29	0.335	4.62 ± 0.31	4.64 ± 0.27	0.802
HR (bpm)	84.17 ± 7.91	79.33 ± 8.15	0.006 **	65.18 ± 9.27	62.46 ± 9.32	0.177	78.25 ± 7.81	74.70 ± 7.97	0.040 *

ART, assisted reproductive technologies; SBP, systolic blood pressure; DBP, diastolic blood pressure; MAP, mean arterial pressure; PP, pulse pressure; cSBP, central systolic blood pressure; cDBP, central diastolic blood pressure; cPP, central pulse pressure; PWV, pulse wave velocity; HR, heart rate. ^1^ A total of 40 ART study participants were included in the analysis. Data are presented as mean ± SD if normally distributed and as median (IQR) if non-normally distributed. * *p*-value < 0.05, ** *p*-value < 0.01.

## Data Availability

The original contributions presented in this study are included in the article. Further inquiries can be directed to the corresponding author.

## Abbreviations

The following abbreviations are used in this manuscript:

24 h ABPM	Twenty-four hour ambulatory blood pressure monitoring
ART	Assisted reproductive technologies
BMI	Body mass index
cDBP	Central diastolic blood pressure
cPP	Central pulse pressure
cSBP	Central systolic blood pressure
DBP	Diastolic blood pressure
HR	Heart rate
ICSI	Intracytoplasmic sperm injection
IQR	Interquartile range
IVF	In vitro fertilization
KIDMED	Mediterranean diet quality index for children and adolescents
MAP	Mean arterial pressure
MEDAS	Mediterranean diet adherence score
MET	Metabolic-equivalent
PP	Pulse pressure
PWV	Pulse wave velocity
SBP	Systolic blood pressure
SD	Standard deviation
WHO	World Health Organization
